# Affibody-based targeting agent ^131^I-YZ_HER2: V2_ for HER2-positive ovarian cancer xenografts

**DOI:** 10.3389/fmed.2025.1507596

**Published:** 2025-07-09

**Authors:** Hongyu Hu, Xianwen Hu, Fangming Li, Guanlian Wang, Jiong Cai

**Affiliations:** Department of Nuclear Medicine, Affiliated Hospital of Zunyi Medical University, Zunyi, China

**Keywords:** human epidermal growth factor receptor 2, affibody, 131 I, radionuclide targeted therapy, HER2

## Abstract

**Background:**

The human epidermal growth factor receptor 2 (HER2) affibodies are multifunctional tools that, when labeled with radioactive isotopes, hold significant potential for the diagnosis and treatment of tumors exhibiting HER2 overexpression. This research focuses on the development of ^131^I-labeled HER2 affibodies as targeted radionuclide therapy agents (TRNT) for HER2-positive Ovarian carcinoma.

**Methods:**

The YZ_HER2: V2_ affibody targeting HER2 was synthesized through genetic recombination. It was labeled with ^131^I by the chloramine T method, and its radiochemical purity and stability were evaluated *in vitro*. The normal mice were subjected to a study on the pharmacokinetic characteristics of ^131^I-YZ_HER2: V2_. An assessment was conducted on the uptake in tumors, biological distribution, and potential for therapeutic use of ^131^I-YZ_HER2: V2_ using a HER2-positive SKOV-3 nude mouse model. The HER2-negative ID-8 mouse model was used as a negative control.

**Results:**

^131^I-YZ_HER2: V2_ was easily prepared, and the non-decayed corrected yield of ^131^I-YZ_HER2: V2_ affibody molecular probe was 96.06% ± 1.26%, showing good stability within 6 h in both normal saline (NS) and fetal bovine serum (FBS). The affinity of ^131^I-YZ_HER2: V2_ was 32.9 nmol/L by cell binding assay. Scintigraphy revealed rapid uptake of the tracer in HER2-positive tumors. The retention of radioactive metabolites in the stomach, kidney, and bladder indicates that radioactive metabolites are mainly excreted through the gastrointestinal tract and urinary system. No substantial radioactive accumulation was observed in the heart, liver, lungs, or muscle tissue. Notably, significant renal retention was also evident based on *in vitro* biological distribution analysis. Tumor accumulation, extended retention, and advantageous distribution were observed in mice with HER2-positive tumors. Mice treated with ^131^I-YZ_HER2: V2_ showed reduced tumor growth and prolonged survival. In the negative control group, there was no obvious aggregation and inhibition of tumors, and radioactive uptake in the kidney and gastrointestinal tract was also observed.

**Conclusion:**

^131^I-YZ_HER2: V2_ has the potential to be explored as a new method for TRNT in HER2-positive ovarian cancer.

## Introduction

Ovarian cancer, with a mortality rate second only to cervical cancer among malignant tumors of the female reproductive tract, is categorized into various subtypes depending on the levels of molecular biomarkers expressed. These biological indicators include the receptors for estrogen and progesterone, as well as the human epidermal growth factor receptor 2 (HER2) (1–3). The presence of HER2 gene amplification or high levels of HER2 protein can be observed in approximately 25 to 30% of ovarian malignancies (4). Overexpression of HER2 can inhibit cell apoptosis, induce angiogenesis and lymphatic angiogenesis, improve cell motility and enhance tumor invasion and metastasis, thus promoting cell growth and proliferation and tumorigenesis. Most studies have consistently shown a significant association between the existence of HER2 protein and an adverse prognosis in ovarian cancer, along with its influence on tumor sensitivity to chemotherapy and biological therapy. As a result, HER2 emerges as a crucial molecular target for further exploration into immunotherapeutic strategies (5, 6). Over the past few years, HER2-targeted therapy has become the favored strategy for the management of patients exhibiting HER2-positive status (7). While targeted therapy for HER2 is effective, the majority of patients in certain subgroups still experience disease progression due to acquired drug resistance. Many researchers are now exploring radioimmunotherapy (RIT) and other immunotherapeutic approaches, aiming to discover innovative strategies and methodologies to comprehensively address ovarian carcinoma (8).

Targeted radionuclide therapy (TRNT) is gaining popularity as a promising approach for treating cancer, particularly in individuals with advanced stages of the disease (9). Radionuclide ^131^I with carrier labeling is used for imaging or treatment of thyroid cancer nodules and accumulates in lesions through physiological uptake or binding to tumor cells with high specificity and affinity. These vectors include antibodies, fragments of antibodies, peptides, as well as other small molecules like affibodies (10). Consequently, TRNT delivers targeted irradiation to tumors without harming surrounding healthy tissues. When the significance of the toxicity associated with external beam radiotherapy is taken into account, TRNT emerges as the favored method for managing disseminated malignancies. Based on the dosimetric verification of Monte Carlo simulation and preclinical data, TRNT is significantly superior to traditional EBRT in terms of accuracy, safety and treatment potential for metastatic diseases through the dual mechanism of molecular targeting and local radiotherapy, especially for MCC and other solid tumors with rich blood vessels and easy to metastasis (11). In the past few years, scientists have utilized various radioactive substances to develop specialized medications for addressing ovarian carcinoma in patients with HER2-positive status. These include emitters of *β*-particles such as lutetium-177 (^177^Lu), rhenium-188 (^188^Re), and iodine-131 (^131^I), as well as emitters of Auger electrons like indium-111 (^111^In). Representative radiopharmaceuticals from this development include ^177^Lu-Dota-Trastuzumab, ^177^Lu-Pertuzumab, ^177^Lu-CHX-A”-DTPA- ABD-(Z_HER2:342_)2, ^188^Re-Z_HER2: V2_, and ^111^In-DTPA-trastuzumab (12–15). The physical characteristics of ^177^Lu *γ*-photons (208 keV, 11% abundancy) are ideal for imaging and also from a radiation safety point of view. Because of the range of *β*-particles, ^177^Lu may have an advantage in treating small lesions (16). When released from the antibody, the metallic radionuclides ^177^Lu are partly incorporated into the mineral bone, which may lead to excessive radiation to the bone marrow (17). Compared with ^177^Lu, ^131^I is the most extensively used radionuclide in TRNT because of its availability, its ease for chemical conjugation, and its ability to perform *γ* imaging and *β* therapeutic studies with the same biological vector (18, 19).

The affibody molecular protein framework is obtained from the Z-binding domain found in Staphylococcus A protein’s IgG binding domain. This particular domain consists of 58 amino termini and exhibits a triple helix structure, often referred to as an “engineered antibody” (20). Compared to monoclonal antibodies and their fragments, affibody molecules stand out due to their small molecular weight (7 KDa), exceptional specificity, strong affinity for antigens, and remarkable ability to penetrate tumors and is well below the glomerular filtration threshold, thus rapidly passing through the glomerular filtration to the urine. These attributes position them as prime candidates for the development of TRNT drugs (12, 15, 21–24). To achieve this objective, our aim is to synthesize a significant amount of ^131^I-labeled YZ_HER2: V2_ and assess its efficacy as a TRNT agent for ovarian carcinoma with HER2-positive expression.

## Materials and methods

### Materials

^131^I was acquired from Beijing Atomic High-tech Co., LTD. (Beijing, China), Chloramine T was obtained from Tianjin Kemiou Chemical Reagent Co., LTD. (Tianjin, China), citric acid was purchased from Sigma-Aldrich Company of the United States, phosphate buffer (PBS;pH 7.4) was purchased from Guiyang Yunyan Jumei Laboratory Reagent (Guiyang, China), Ni Agarose 6FF and Q-Agarose FF were procured from Beijing Solarbio Technology Co., LTD. (Beijing, China). Chongqing Bo Maddison Biocell Center supplied ID-8 (HER2-negative) and SKOV-3 cells (HER2-positive). Agilent Technologies supplied Instant Thin Layer chromatography silica gel (iTLC-SG) paper while GE Healthcare supplied NAP-5 size exclusion column. Isoflurane (1–3% [inhaled]) was purchased from Shenzhen RWD Life Science Co., Ltd. Balb/c nude mice (6 weeks of age, 18-20 g, female), healthy Balb/c mice (4–6 weeks of age, 18–22 g, female) were obtained from Enschville Laboratory Animal Technologies, Inc. Animals were housed in standard conditions: In the absence of specific pathogens (SPF) environment, use ventilated cages, with environmental enrichment, 12 h of light/dark cycle, a controlled temperature and to access to food and water. The GC-2010 gamma radiation counter was purchased from ZONKIA at the University of Science and Technology of China in Hefei, China. Additionally, the Infinia V Hawkeye 4 SPECT/CT imaging system was acquired from GE Healthcare located in Chicago, Illinois, USA.

### Preparation of HER2 affibody protein

*E. coli* BL21 cells were subjected to a transformation procedure using the plasmid pET22b (+), which contained a gene fragment encoding HER2-targeted biophiles. The amino terminal has tyrosine and the amino acid sequence is MAYHEHEHEA ENKFNKEMRN AYWEIALLPN LTNQQKRAFI RSLYDDPSQS ANLLAEAKKL NDAQ. The bacterial cells were grown in LB broth supplemented with kanamycin (50 μg/mL) at 37°C and stimulated for protein expression using IPTG (1 mmol/L). After the completion of harvest, ultrasonic treatment was employed to lyse the cells, followed by centrifugation for the removal of cellular debris. Subsequently, the resulting clarified cell lysate underwent heat treatment at a temperature of 60°C for a duration of 10 min in order to induce precipitation of a fraction of the inherent *E. coli* protein and filtration using a 0.22 μm filter. The affibody was isolated using immobilized metal affinity chromatography (IMAC, Ni Agarose 6FF) and subsequently purified through anion exchange chromatography (Q-Agarose FF).

The affibody underwent analysis utilizing sodium dodecyl sulfate-polyacrylamide gel electrophoresis (SDS-PAGE) and matrix-assisted laser desorption/ionization tandem time-of-flight mass spectrometry (Maldi-TOF/TOF).

### Prepared and characterized ^131^I-YZ_HER2: V2_

Add 200 μL of YZ_HER2: V2_ solution (1 mg/mL in PBS, pH 7.4) into an EP tube, followed by gentle mixing with 100 μL of Na^131^I (3.7 GBq/mL). Subsequently, introduce 80 μL of chloramine T solution (1 g/L). Incubate the mixture at room temperature for 10 min, occasionally shaking, and then quench the reaction by adding 40 μL of Na_2_S_2_O_3_ (0.25 mol/L). Thereafter, purify the reaction mixture using a NAP-5 column and employing PBS as the eluent, through size-exclusion chromatography.

The determination of the labeling efficiency of the molecular probe ^131^I-YZ_HER2: V2_ was performed utilizing reverse-phase high-performance liquid chromatography (RP-HPLC). In essence, the analysis was conducted utilizing an LC-16 HPLC system from Shimadzu (Suzhou, China) equipped with a UV–visible detector (*λ* = 260 nm) and a radioactivity flow detector from ECKERT & ZIEGLER Radiopharma (Berlin, Germany). A WondaSil C18-WR column (5 μm, 100 Å, 4.6 I. D × 150 mm, Cat. No.5020–39,031, Suzhou, China) was employed for the analysis. The identification conditions were as follows: (1) Application of a C18 reverse-phase column. (2) Mobile phase A consists of acetonitrile with 0.1% trifluoroacetic acid, gradually increasing from 5 to 80%. Mobile phase B, on the other hand, is composed of water with 0.1% trifluoroacetic acid and gradually decreasing from 95 to 20%. (3) The rate of liquid flow is 1 mL/min. (4) UV detector wavelength set at 260 nm. (5) Sample run time set to 20 min. The radioactive chemical purity (RCP) of ^131^I-YZ_HER2: V2_ was determined using HPLC, and rapid analysis was conducted using instant thin-layer chromatography (ITLC). The ITLC technique utilized Agilent’s ITLC-paper along with a mobile phase consisting of citrate buffer (pH = 4.5). ^131^I-YZ_HER2: V2_ was incubated at room temperature (approximately 37°C) in both normal saline (NS) and fetal bovine serum (FBS) to assess its *in vitro* stability. The RCP was evaluated using the aforementioned ITLC method within 8 h.

### Cell lines

The SKOV-3 and ID-8 cells were incubated in a 5% CO_2_ incubator at 37°C for 2 to 3 days, using DMEM medium supplemented with 10% fetal bovine serum and 1% penicillin–streptomycin. Once the cell fission rate reached approximately 90%, the initial culture medium was discarded, followed by two washes with PBS. Subsequently, a suitable solution of trypsin (0.25%) containing EDTA (0.02%) was added to detach cells from the flasks. When the cells shrank back and became round, supplemented DMEM was added into the complete culture medium to stop digestion, the wall of the bottle was gently blown with a sterile pipette, the cells attached to the wall were blown off and transferred to the centrifuge tube. After being subjected to centrifugation at 217 × g for a duration of 5 min, the supernatant was removed. Subsequently, DMEM was introduced into the complete culture medium and thoroughly blended through blowing and mixing processes. Subsequently, cells were introduced into the culture bottle at a concentration ratio of 1:3 for passage.

### Specific binding and cellular uptake

SKOV-3 cell line was selected the positive control and ID-8 cell line as negative control. They were inoculated into 24-well plates (2 × 10^5^/well, 1 mL) and cultured at 37°C overnight. When the cells had attached as a monolayer, the original medium was discarded, the cells rinsed twice with PBS, and fresh serum-free medium (DMEM) was added. Freshly prepared ^131^I-YZ_HER2: V2_ at different concentrations (0.52, 2.59, 12.97, 64.86, 162.16, 405.43 nM, *n* = 3) were added to each well. After subjecting the cells to incubation at a temperature of 4°C for a duration of 2 h, the medium was extracted and the cells were rinsed thrice with cold PBS. Subsequently, lysis was initiated by exposing them to 1 M NaOH. The resulting lysates obtained from the cells were gathered and quantified using a gamma counter. Using GraphPad Prism 9 software through nonlinear fitting (total number of unit points and non-specific binding) calculate equilibrium dissociation constant (equilibrium dissociation constant, Kd).

SKOV-3 cells were inoculated in culture dishes (10^6^/dish, 2 mL) and cultured overnight at 37°C to form a monolayer. Eliminate the medium, wash twice with PBS, and introduce a fresh serum-free medium. ^131^I-YZ_HER2: V2_ was added to the solution at a concentration of 32.9 nM and incubated at a temperature of 37°C. At a predetermined time point (1, 2, 4, 8, 12, and 24 h after the start of incubation), a set of petri dishes (*n* = 3) were extracted. The liquid portion was gathered while the cells underwent three rounds of cold PBS rinsing. The washing medium was mixed with the supernatant previously collected, which represented unbinding. Then, the cells were exposed to a chilled urea buffer (4 M urea, 0.2 M glycine, pH 2.5) for a period of 5 min, and the cells were washed 3 times to collect the urea buffer, which represents membrane binding. Finally, cell lysis was accomplished using a solution containing 1 mol/L NaOH, and the culture dish was cleaned three times, and the cell lysate and cleaning solution were collected, which represented the cell internalization. The gamma counter quantifies the amount of gamma-emitting radionuclides in a sample and determines the proportion of radioactivity bound to the membrane and internalized over time.

### Animal models

All experiments involving animals have received ethical approval from the Ethics Committee of the Affiliated Hospital of Zunyi Medical University (Approval number, KLLYA-2021-019). Euthanasia by cervical dislocation was performed 4 weeks after the end of the protocol and had been planned in case of poor tolerance but was not required. The mice were kept in adequately ventilated cages with filter tops and given unrestricted availability to a standard diet and water. SKOV-3 and ID-8 cells were cultured to the exponential growth phase and were then re-suspended in PBS buffers after trypsin digestion. SKOV-3 and ID-8 cells (5 × 10^6^, 100 μL) were implanted into the right axillary region of 21 female Balb/C mice aged 6–8 weeks, respectively, and tumors were formed within about 2 weeks. The size of the tumor was assessed using a vernier caliper. Once the tumor volume reached approximately 1 cm^3^, it was utilized for conducting a biological distribution analysis and scintigraphy.

### Pharmacokinetics of ^131^I-YZ_HER2: V2_

A pharmacokinetic study of ^131^I-YZ_HER2: V2_ was conducted on 33 Balb/c mice, aged between 6 and 8 weeks, with an approximate average weight of 18.0 ± 1.6 g, procured from Ensville Co., Ltd., Chongqing, China. Each mouse received an intravenous injection in the tail vein of 0.74 MBq of ^131^I-YZ_HER2: V2_, equivalent to 5 μg of YZ_HER2: V2_ in 100 μL of PBS. Blood samples were drawn at specific time points post-injection: 0, 10, 15, 30 min, and 1, 2, 4, 8, 24, 48, and 72 h, with three mice sampled at each interval. At these respective times, a blood sample of 0.2 mL was obtained post-enucleation of the eyeball, placed into test tubes, and the radioactivity of each sample was assessed using a *γ*-counter. The level of radioactivity in the bloodstream was quantified as the ratio of the administered dose to tissue weight (%ID/g). A time-radioactivity curve of blood was generated. Pharmacokinetic parameters were calculated using DAS 2.0 software from Shanghai, China, and a two-compartment model was utilized to assess the half-life of ^131^I-YZ_HER2: V2_ in the bloodstream.

### Biodistribution study

The injection of approximately 1.85 MBq (10 μg)of ^131^I-YZ_HER2: V2_ was administered into the lateral tail vein of mice bearing SKOV3 tumors. Animals were euthanized at 1, 6, 24, and 72 h after injection. To minimize animal suffering, humane euthanasia was performed promptly via CO_2_ inhalation. Following euthanasia, animals were placed in lead-lined containers for 3 days and subsequently cremated after surface contamination measurements confirmed absence of residual radioactivity. Samples including blood, tumor, cardiac tissue, hepatic tissue, splenic tissue, pulmonary tissue, renal tissue, cerebral tissue, muscular tissue, femoral bone marrow, gastric mucosa and duodenal mucosa were gathered. A *γ*-counter measures the radioactive count of a sample. The quantification of biological distribution was represented as the ratio of injected dose to tissue weight (%ID/g).

### Scintigraphy and immunohistochemistry analyses

SKOV-3 ovarian cancer-containing nude mice were divided into two groups, one group of 3 days in advance take feed a large excess of non-radioactive Iodine (1% NaI) was used to block uptake of radioactive I into the thyroid gland, with isoflurane anesthesia (4–5% induction, maintain at 1–3%). Each group was injected with about 1.85 MBq of ^131^I-YZ_HER2: V2_ (YZ_HER2: V2_ content was about 10 μg) through different administration routes: caudal vein, subcutaneous and abdominal. Scintigraphy was conducted at intervals of 1, 3, 6, 24, 48, and 72 h post-injection. Image acquisition parameters were 128 × 128, magnification was 2.5 times, and radiation count was 200.

### Therapeutic efficacy

The established SKOV3 tumor-bearing mice were randomly assigned to three groups, each comprising seven mice. PBS (100 μL), YZ_HER2: V2_ (100 μL, equivalent to 20 μg) dissolved in sterile water, and ^131^I-YZ_HER2: V2_ (3.7 MBq, 100 μL), corresponding to 20 μg of YZ_HER2: V2_, were administered in each group. Treatment consisted of every 5 days, a total of 5 times. Tumor volume in the mice was measured after each treatment. The nude mice were euthanized when the tumor volume was greater than 2000 mm^3^, weight loss was 20–25%, tumor ulceration or necrosis occurred, or other reasons related to animal ethical violations occurred.

### Statistical analysis

SPSS 18.0 software was used. Data that followed a normal distribution were expressed as mean ± standard deviation (^−^x ± s). The independent samples t-test was utilized to compare two groups, while One-Way ANOVA was used for comparing multiple groups. A significance threshold of *p* < 0.05 was chosen, with levels of significance indicated by *p < 0.05; **p < 0.01; ***p < 0.001.

## Results

### Quality control and stability of ^131^I-YZ_HER2: V2_

**Figure 1:**
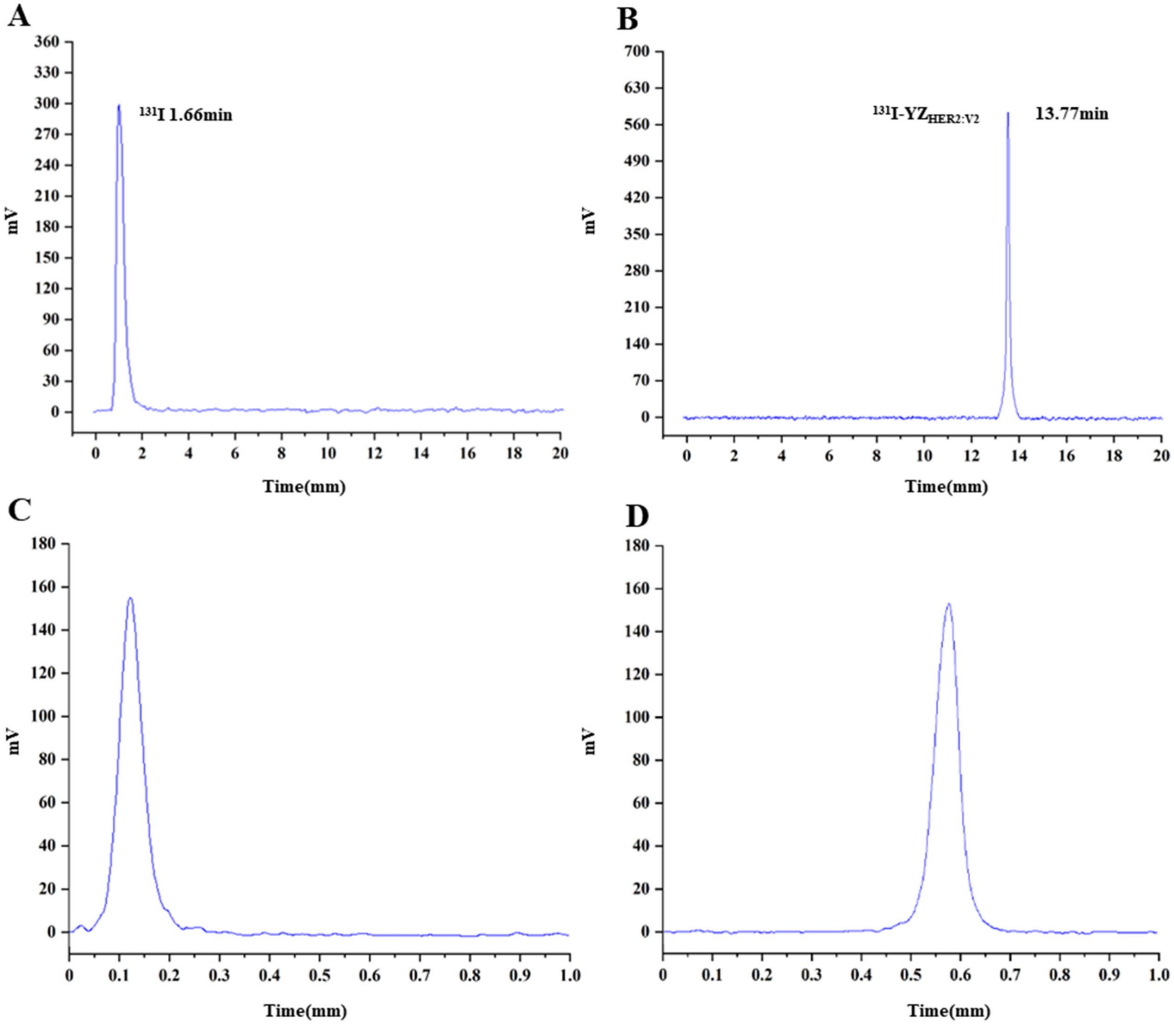
Characterization and stability assessment of ^131^I-YZ_HER2: V2_ were conducted. HPLC analysis was performed on ^131^I-YZ_HER2: V2_ **(A)** and ^131^I **(B)**. The results of instant thin-layer chromatography (ITLC) showed the presence of ^131^I-YZ_HER2: V2_ **(C)** and ^131^I **(D)**.

**Figure 2:**
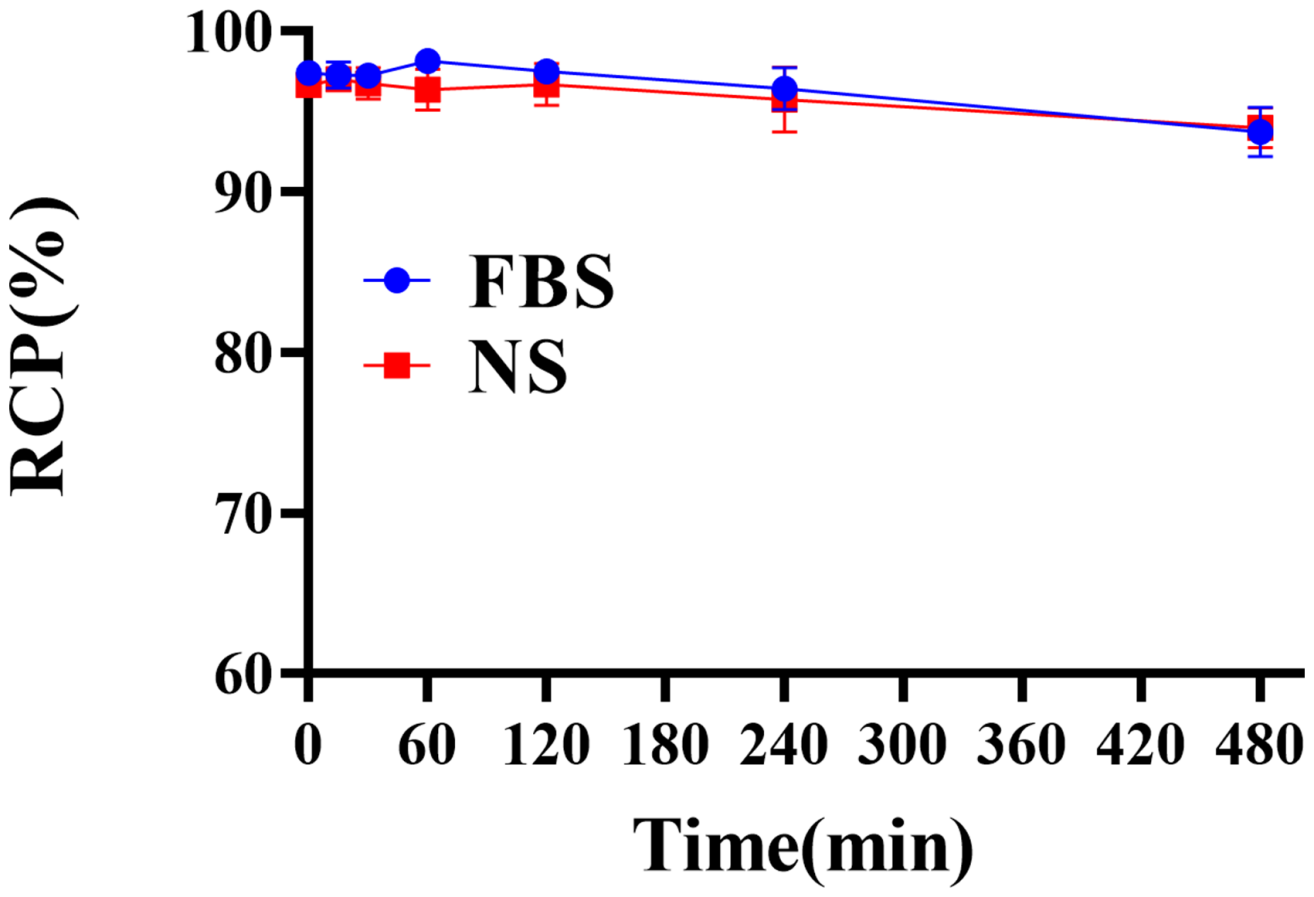
Assessing the stability of ^131^I-YZ_HER2: V2_ in a solution of normal saline (NS) at room temperature and fetal bovine serum (FBS) at 37°C for up to 8 h.

The successful radiolabeling of YZ_HER2: V2_ with ^131^I was accomplished using the chloramine-T technique. Characterization of ^131^I-YZ_HER2: V2_ was conducted through HPLC and ITLC. As depicted in (Figures 1A,B), the HPLC analysis of ^131^I-YZ_HER2: V2_ exhibited a solitary radioactive peak with a retention time of 13.77 min, whereas the HPLC outcome for ^131^I demonstrated a solitary radioactive peak with a retention time of 1.66 min. The labeling yield of ^131^I-YZ_HER2: V2_ was found to be high at (96.06% ± 1.26%) (n = 5), eliminating the requirement for further purification in biological experiments. The bacterial endotoxin assay demonstrated a minimal presence of less than 1 EU/mL. ^131^I-YZ_HER2: V2_ exhibited a retention factor ranging from 0 to 0.2 (Figure 1C), while iodine ions had a retention factor ranging from 0.5 to 0.7 (Figure 1D). Furthermore, the stability of ^131^I-YZ_HER2: V2_ was analyzed in both normal saline (NS) and fetal bovine serum (FBS) at room temperature (approximately 37°C) via the ITLC method. The stability of ^131^I-YZ_HER2: V2_ was maintained in both NS and FBS, with a RCP of over 90% achieved within 8 h (Figure 2).

### Pharmacokinetics of ^131^I-YZ_HER2: V2_

**Figure 3:**
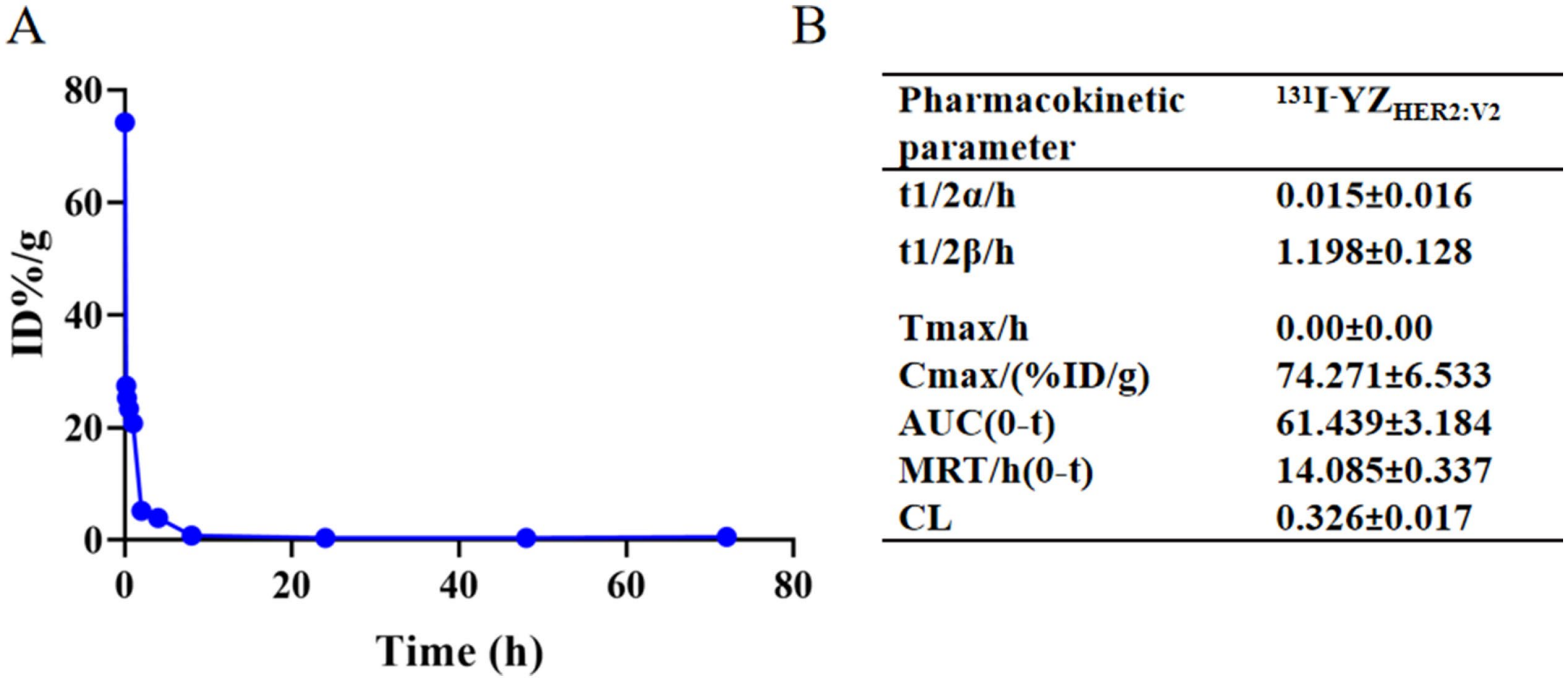
**(A)** Time-concentration profile of the drug ^131^I-YZ_HER2: V2_; **(B)** Pharmacokinetic characteristics of ^131^I-YZ_HER2: V2_.

The drug-time curve and pharmacokinetic parameters of ^131^I-YZ_HER2: V2_ are depicted in Figure 3. The administration of ^131^I-YZ_HER2: V2_ intravenously into the mouse tail resulted in an immediate peak observed at zero Tmax value (Tmax = 0). The distribution half-life (T1/2α) was found to be approximately 0.015 ± 0.016 h, while the elimination half-life (T1/2β) was estimated to be around 1.198 ± 0.128 h. A maximum concentration achieved (Cmax) of roughly (74.27 ± 6.53)% ID/g was noted, along with an average residence time of about 14.9 ± 0.337 h and a clearance rate of approximately (0.326 ± 0.02)% ID/g/h.

### Binding specificity and cellular uptake

**Figure 4:**
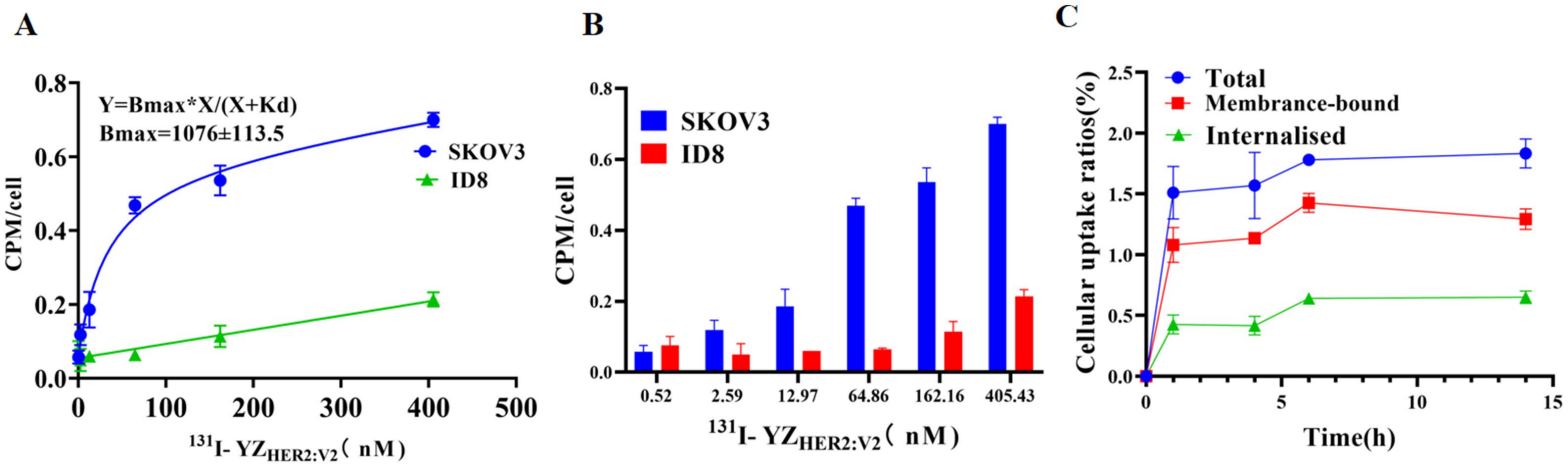
Binding specificity and cellular uptake. **(A)** Detection of ^131^I-YZ_HER2: V2_ using Affibody and its application in the identification of HER2-positive cells. **(B)** Examining the impact of different concentrations of ^131^I-YZ_HER2: V2_ on SKOV3 and ID8 cell lines (*n* = 3). **(C)** Internalization and absorption of ^131^I-YZ_HER2: V2_ in SKOV3 cells.

At all concentration points, the radioactivity of ^131^I-YZ_HER2: V2_ in SKOV-3 cells was found to be significantly greater than that observed in ID-8 cells (*p* < 0.05) (Figures 4A,B). ^131^I-YZ_HER2: V2_ has an affinity for SKOV-3 cells with KD values of about (32.9 ± 0.69) nM. Cell uptake studies showed that SKOV-3 cells rapidly took up ^131^I-YZ_HER2: V2_, and the uptake rate was (1.51 ± 0.22)% at 1 h, and then slowly increased, and the uptake rate was (1.78 ± 0.35)% at 6 h. The internalization of ^131^I-YZ_HER2: V2_ by SKOV3 cells increased over time, with (3.78 ± 0.25)% of total cell-binding radioactivity internalized after 14 h of incubation (Figure 4C).

### Biodistribution in SKOV-3 nude mice

**Figure 5:**
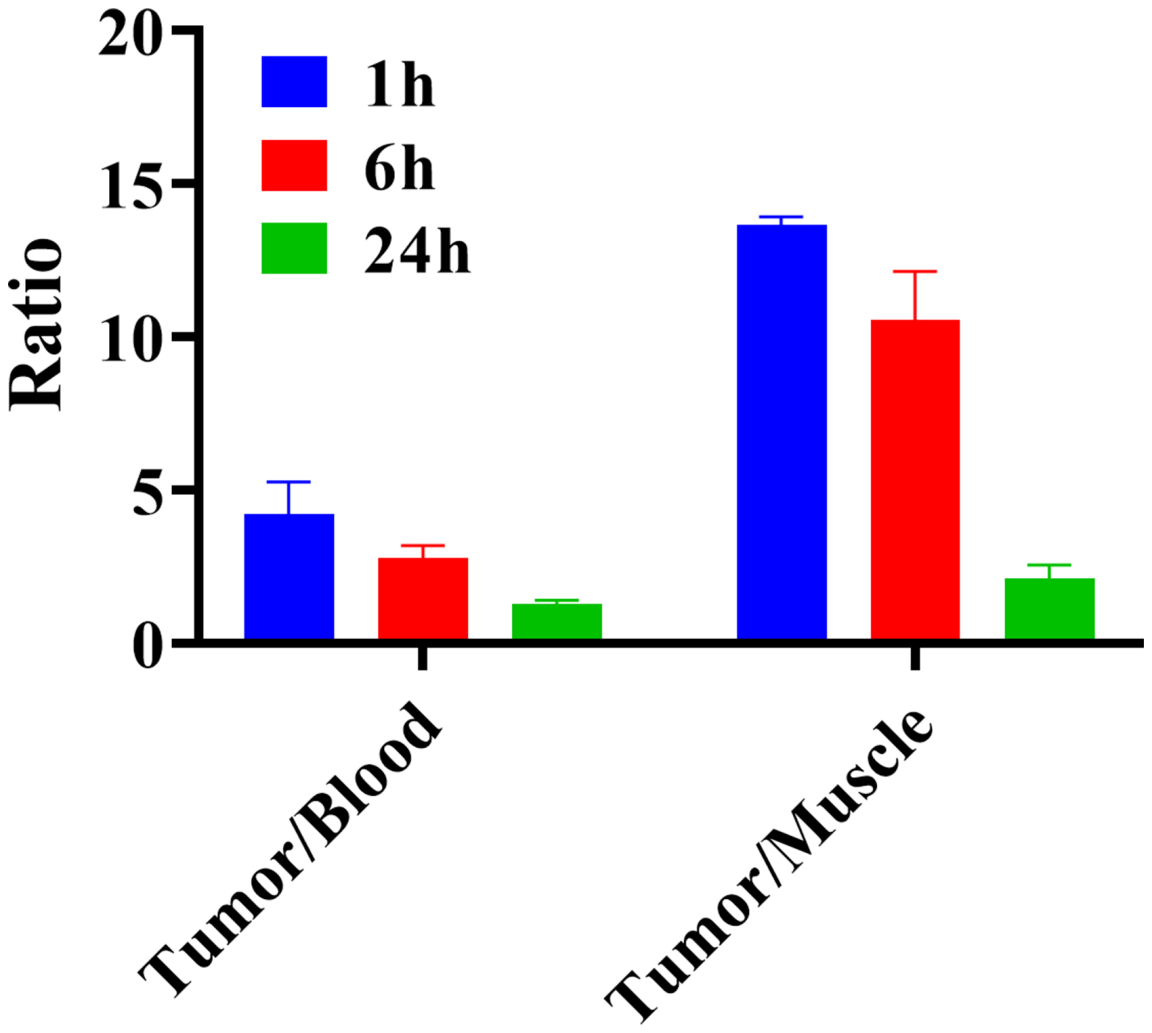
Ratio of ^131^I-YZ_HER2: V2_ uptake to blood and muscle in mice bearing SKOV3 tumors (n = 3).

The tumor uptake was (27.29 ± 0.55) %ID/g at 1 h and (1.75 ± 0.44) %ID/g at 24 h after injection of ^131^I-YZ_HER2: V2_. The biological distribution data for ^131^I-YZ_HER2: V2_ in SKOV-3 nude mice are shown. An hour post-injection, variations in the ratio between tumor and blood were detected. The tumor-to-muscle ratio at 1 h was significantly higher than at 6 and 24 h, as depicted in Figure 5.

### Scintigraphy of SKOV-3 nude mice

**Figure 6:**
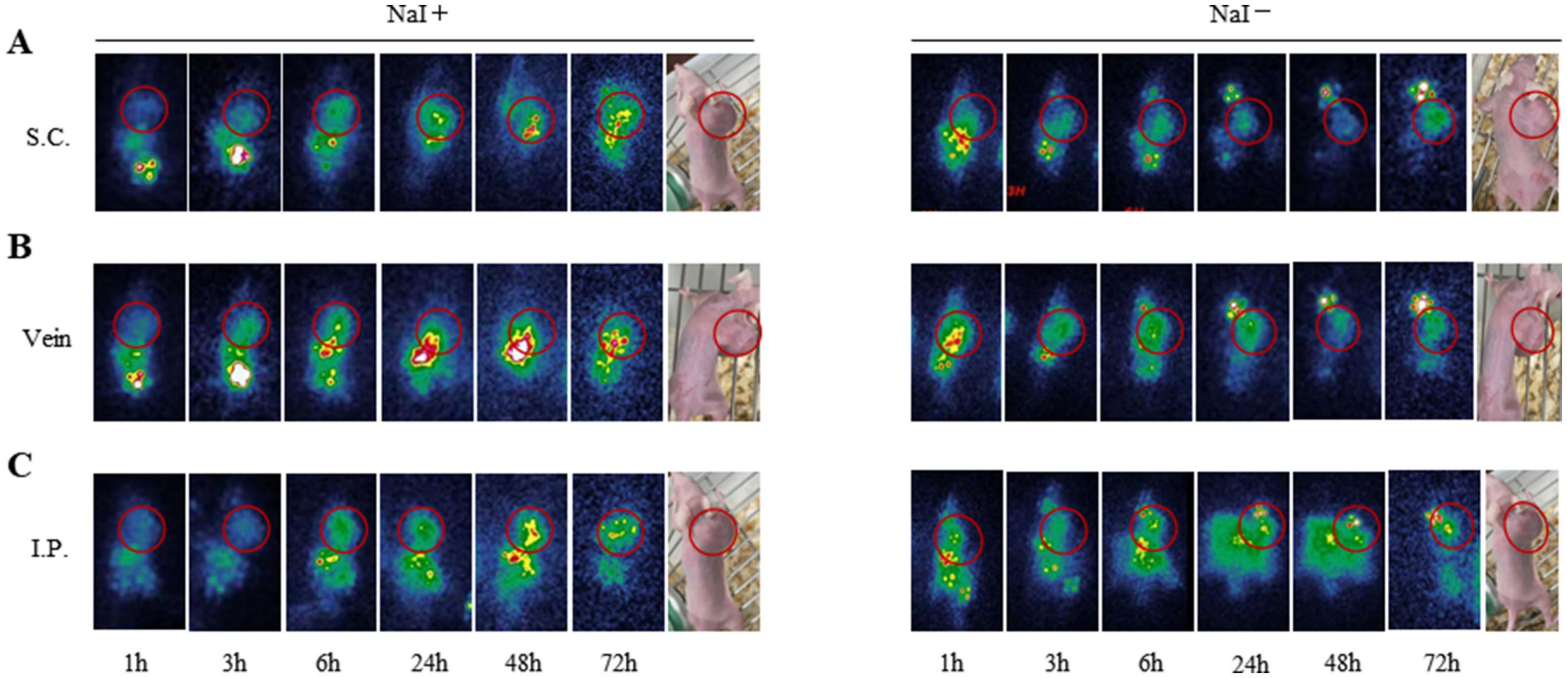
Scintigraphy of SKOV3 tumor bearing mice in different administration routes and imaging time. **(A)** subcutaneous injection **(B)** intravenous injection **(C)** intraperitoneal injection of 1 h, 3 h, 6 h, 24 h, 48 h, 72 h imaging. (NaI-/NaI+).

The schematic diagram for scintigraphy is presented in Figure 6. After injection of ^131^I-YZ_HER2: V2_ into the tail vein of SKOV-3 ovarian cancer bearing nude mice, the tumor radionuclide concentration was clearly shown. Over time, it becomes evident that intraperitoneal injection offers a more distinct visualization of the tumor. This enhanced clarity is possibly attributed to the peritoneal absorption, rendering it a promising administration route for our subsequent treatment experiments. One hour following the injection of ^131^I-YZ_HER2: V2_, a significant accumulation of radioactive isotopes within the tumor is observed. The detection of radioactivity buildup in the kidneys, bladder, and gastrointestinal tract suggests that ^131^I-YZ_HER2: V2_ primarily exits the body through the urinary and digestive systems, aligning with its natural distribution. In the group where the thyroid uptake of radioactive I is blocked by a superabundance of non-radioactive I, no radioactive isotopic accumulation was detected in the thyroid. This minimizes the risk of thyroid damage and further enhances labeling efficiency. By manually delineating the tumor and lower limb (depicting the level of radioactivity in muscles), we obtained the average radiation count per unit volume. The results show a decreasing ratio with time, in line with the biological distribution pattern. However, no radionuclide concentration was observed in ID8 tumors, as shown in Supplementary Figure S4.

### Therapeutic results

**Figure 7:**
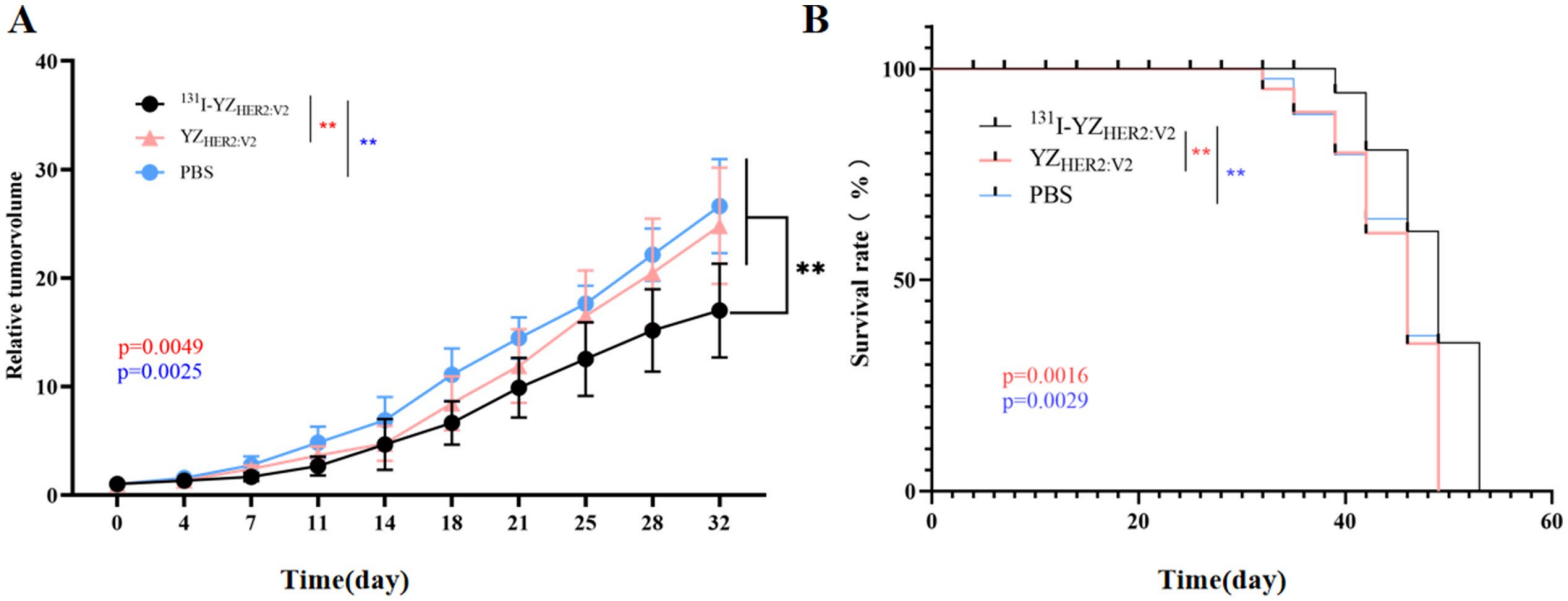
*In vivo* therapeutic efficacy of ^131^I-YZ_HER2: V2_ in HER2-positive tumors. **(A)** Relative tumor volume. **(B)** survival rate. The red asterisk (*) indicates a comparison between the experimental group ^131^I-YZ_HER2: V2_ and the control group YZ_HER2: V2_, while the blue asterisk (*) represents a comparison between ^131^I-YZ_HER2: V2_ and the blank group PBS.

After 53 days of treatment, we observed similar growth patterns in the relative tumor volume of the YZ_HER2: V2_ group (24.80 ± 5.36) times and the PBS group (26.62 ± 4.33) times, with no significant differences in their relative tumor volumes, both of which exceeded that of the ^131^I-YZ_HER2: V2_ group (17.00 ± 4.32) times. Tumors in mice subjected to YZ_HER2: V2_ and PBS treatments exhibited exponential growth, while those receiving ^131^I-YZ_HER2: V2_ treatment experienced a delay in tumor growth, as illustrated in Figure 7A. In addition, as shown in Figure 7B, the survival time of ^131^I-YZ_HER2: V2_ group was 3 days longer than that of YZ_HER2: V2_ group and PBS group, with a statistically significant difference (*p* < 0.05), indicating that ^131^I-YZ_HER2: V2_ related tumor shrinkage can affect survival.

## Discussion

In the clinical trials that were conducted, HER2 affibody was labeled using diagnostic radionuclides including fluorine-18 (^18^F), gallium-68 (^68^Ga), technetium-99 (^99m^Tc), and ^111^In to evaluate tumor targeting, biological distribution, blood clearance, dosimetry, and safety (25–28). The successful clinical transformation of affibody targeting HER2-positive tumors has prompted efforts to create additional TRNT drugs targeting HER2 for the treatment of cancer. In 2007, Tolmachev et al. (15). were the first to introduce the application of CHX-A”-DTPA as a chelating agent in order to attach ^177^Lu to the affibody Z_HER2:342_ for labeling purposes. The findings indicated that the administration of ^177^Lu-CHX-A”-DTPA-ABD-(Z_HER2:342_)_2_ effectively inhibited the growth of SKOV-3 cells characterized by high HER2 receptor expression. The survival of nude mice treated with ^177^Lu-CHX-A “-DTPA-ABD -(ZHER2:342)2 was significantly longer than that of the control group injected with PBS and the blocking group injected with ^177^Lu-CHX-A” -DTPA-ABD -(ZHER2:342)2. In the control group, 70% of mice developed tumors between 36 and 62 days (median 43 days) and died due to tumor growth between 67 and 104 days (median 67 days). None of the mice in the 21.6 MBq treatment group developed tumors, and the survival rate was 100%, which was significantly different from that in the control group (*p* < 0.001). However, its clinical translation is limited due to its elevated radioactive retention in blood and bone marrow. In this research, we assessed the efficacy of YZ_HER2: V2_ labeled with ^131^I as a TRNT agent for managing transplanted tumors of HER2-positive breast cancer in mice. ^131^I is an ideal choice for radionuclide therapy and SPECT imaging due to its emission of both *β*-particles and *γ*-rays. To date, HER2-positive ovarian cancer has not been treated with ^131^I-labeled HER2 affibody. Altai et al. (22) attached different amino acid sequences to the carboxyl terminus, labeled these modifiers with ^188^Re, and compared them *in vitro* and *in vivo*. The results showed that the tumor uptake of ^188^Re-Z_HER2: V2_, a molecular probe with GGGC sequence at the carboxyl terminus, was significantly higher than that of any organ tissue (including liver and kidney) at 4 h after injection. This molecular probe has a high yield and maintains a high affinity for HER2. ^188^Re-Z_HER2: V2_ provides highly efficient targeting of HER2-expressing xenografts, rapid blood clearance, and low renal and bone uptake. Preclinical studies have shown that ^188^Re-labeled YZ_HER2: V2_ specifically binds to SKOV-3 cells and is rapidly cleared from the bloodstream. Four hours after injection, radioactive uptake in the tumor exceeded that in the kidneys, with scintigraphy further emphasizing the tumor as the primary site of radioactive accumulation. Yang et al. (29) and Hu et al. (30) used a rapid and simple method to directly label HER2: V2 with ^99m^Tc. This method is simple, rapid, and has a high labeling rate and high radiochemical purity. The radiolabelled probe showed good HER2 tumor targeting, rapid blood clearance, low gastric and salivary gland radioactivity, low renal radioactivity, and low hepatobiliary excretion. HER2 affibody YZ_HER2: V2_ labeled with ^131^I was used in current study. Additionally, ^131^I can be efficiently coupled with YZ_HER2: V2_, which has tyrosine residues, using the chloramine-T method. At present, different methods are commonly used to label iodine with radioisotopes, including chloramine-T, iodogen, and lactoperoxidase techniques (31–33). Furthermore, SGMIB has emerged as a novel technique for radioiodination, offering notable benefits compared to conventional iodination methods. These advantages stem from the gentle reaction conditions, excellent reproducibility, and minimal compromise of biological activity, resulting in enhanced biodistribution and superior tumor uptake of radioiodinated biomolecules (34) (see Table 1).

**Table 1:** Biodistribution of ^131^I-YZ_HER2:V2_ in SKOV3 tumor-bearing mice (%ID/g, x ±s, *n*=3).

Tissues (%ID/g)	Time (1 h)	Time (6 h)	Time (24 h)	Time (72 h)
Blood	6.70±1.80	4.30±1.04	1.84±0.30	0.62±0.18
Heart	3.69±0.14	3.29±0.53	1.52±0.09	1.36±0.14
Liver	3.80±0.19	1.48±0.42	0.86±0.09	0.72±0.07
Spleen	3.74±0.17	3.38±0.08	2.31±0.01	1.87±0.14
Lung	6.26±0.39	4.42±0.29	3.35±0.13	2.64±0.21
Kidney	80.27±3.07	26.30±2.77	23.69±0.70	14.99±1.41
Brain	0.48±0.08	0.32±0.05	0.24±0.03	0.16±0.01
Gastrointestinal	39.72±1.39	31.67±1.75	7.42±1.34	0.58±0.07
Muscle	2.00±0.08	1.14±0.28	1.12±0.17	0.22±0.16
Bone	3.39±0.28	2.35±0.08	1.84±0.01	1.47±0.28
duodenum	8.78±0.03	6.77±0.40	5.05±0.04	0.26±0.07
Tumor	27.29±0.55	11.81±1.17	2.34±0.14	1.749±0.44

Each treatment group received five doses at a dose frequency of 0.1 mCi for the cumulative dose of the treatment described herein. We observed that the YZ_HER2: V2_ labeled with ^131^I exhibited a significantly high RCP and demonstrated satisfactory stability for up to 6 h in an *in vitro* setting. After the administration of ^131^I-YZ_HER2: V2_ via intravenous injection in mice, rapid elimination from the bloodstream is observed, facilitating the acquisition of early diagnostic images characterized by excellent contrast. As expected, HER2-positive tumors became detectable within one hour of administration, whereas minimal accumulation in HER2-negative tumors was observed during the corresponding time period. The T/M ratio of HER2-positive SKOV-3 xenografts was consistently higher compared to the negative controls at all time points. The imaging results of intravenous, subcutaneous and intraperitoneal injection routes showed that the tumors were visualized by all three injection methods, but the animal model injected intraperitoneally had better imaging effect over time. *In vivo* tumor targeting specificity of ^131^I-YZ_HER2: V2_ against HER2-positive tumors was further confirmed by observing a significant increase in tumor uptake during biological distribution experiments conducted 1 h post-injection. Swift elimination from the bloodstream may help reduce radiation exposure to non-target tissues, thereby minimizing potential side effects or toxicity. However, it might lead to insufficient drug concentration in the target tissues, compromising the therapeutic efficacy. As a result, to achieve the desired therapeutic outcome, more frequent dosing or higher infusional doses might be necessary.

The efficacy of ^131^I-YZ_HER2: V2_ in treating HER2-positive tumors was proven through the administration of 5 treatment sessions. The tumor suppressive effect of ^131^I-YZ_HER2: V2_ was attributed to HER2-mediated targeting of ovarian cancer cells for tumor uptake, which was also associated with longer survival. After treatment with ^131^I-YZ_HER2: V2_, the survival time in this group was extended to 53 days, while all mice in the PBS group and the YZ_HER2: V2_ group were euthanized at this time point due to ethical reasons, such as tumor volumes exceeding 2,000 mm^3^, a weight loss of 20–25%, tumor ulceration, or necrosis. The two groups were observed for 53 days, and the ^131^I-YZ_HER2: V2_ group had prolonged cell survival compared with the YZ_HER2: V2_ group and PBS group. Further preclinical studies need longer observation periods to evaluate the duration of delayed tumor growth.

Taken together, the *in vivo* results indicate that ^131^I-YZ_HER2: V2_ exhibits promising efficacy and safety profiles, suggesting its potential as a TRNT agent for ovarian cancer with HER2 overexpression.

The results show that the affinity labeling method is feasible, rapid and highly radiochemically pure. The rapid blood clearance, the good tumor targeting, and the high renal and gastrointestinal radioactivity suggest renal and gastrointestinal excretion of unbound avidin. ^131^I-YZ_HER2: V2_ is a promising probe for the diagnosis and treatment of HER2-overexpressing tumors. In our research, due to the strong binding affinity and effective tumor targeting capabilities of YZ_HER2: V2_, we selected it as a promising candidate for the development of molecular imaging agents and targeted therapy drugs in order to diagnose and treat HRE2-positive cancers. The dosimetric safety of ^131^I-YZ_HER2: V2_ has not been evaluated before treatment, which needs to be further improved in future studies.

## Conclusion

^131^I-YZ_HER2: V2_, synthesized utilizing the Chloramine-T technique, demonstrates a remarkable efficiency in radiolabeling and exceptional stability when tested *in vitro*. The produced ^131^I-YZ_HER2: V2_ demonstrates tumor accumulation, prolonged tumor retention, a high tumor-to-background ratio, favorable biodistribution and therapeutic efficacy in mice bearing HER2-positive tumors. Our findings indicate that ^131^I-YZ_HER2: V2_ exhibits significant potential as a highly promising therapeutic radiopharmaceutical for future clinical applications.

## Data Availability

The original contributions presented in the study are included in the article/Supplementary material, further inquiries can be directed to the corresponding author.
