# Supplementary material for: Affibody-based targeting agent 131I-YZHER2: V2 for HER2-positive ovarian cancer xenografts
Source: Front Med (Lausanne). 2025 Jul 9;12:1507596. doi: 10.3389/fmed.2025.1507596 (PMC12283983; doi:10.3389/fmed.2025.1507596)
Supplement: Supplementary file 1 [file Data_Sheet_1.doc]

**^131^I labeled HER2** **affibody protein for targeted radionuclide therapy of HER2-positive ovarian cancer**

Hongyu Hu***^†^***, Xianwen Hu***^†^***, Fangming Li, Guanlian Wang and Jiong Cai*

*Department of Nuclear Medicine, Affiliated Hospital of Zunyi Medical University, Zunyi, 563002, China;*

***^†^***These authors have contributed equally to this work.

**Correspondence to:* Dr Jiong Cai, Department of Nuclear Medicine, The Affiliated Hospital of Zunyi Medical University, 149 Dalian Road, Huichuan, Zunyi, 563003, P.R. China

E-mail: jiong_cai@163.com


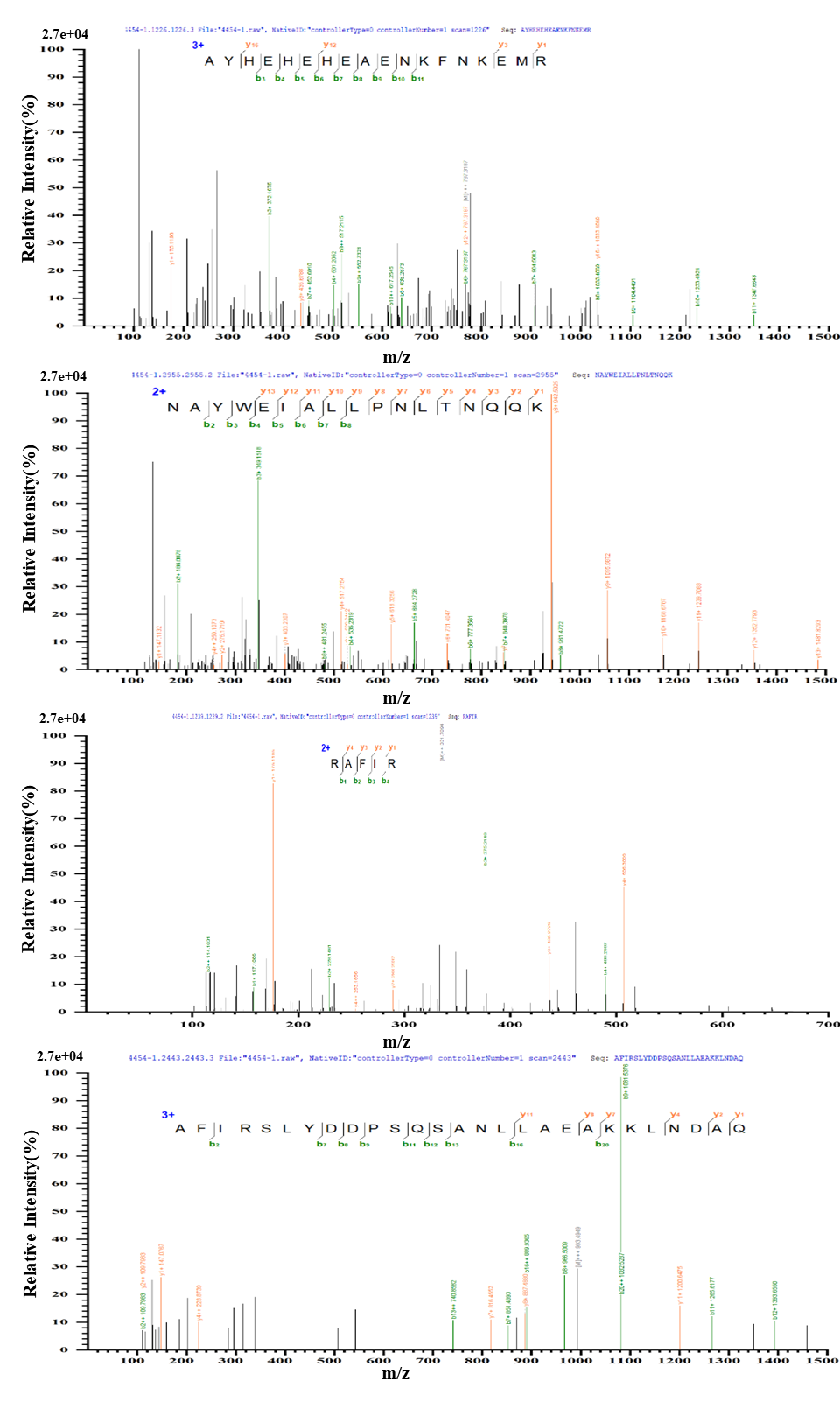


**Figure S1**. Amino acid sequence determination of YZ_HER2:V2_ by matter-assisted laser desorption ionization tandem time-of-flight mass spectrometry (Maldi-TOF/TOF).


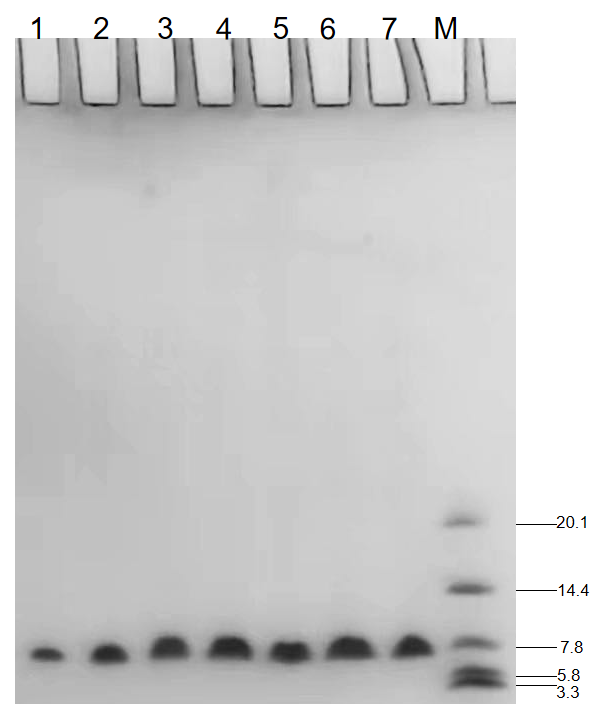


**Figure S2**. An investigation was conducted on the purification process of YZHER2:V2 using sodium dodecyl sulfate and polyacrylamide gel. The Affibody was purified through anion exchange chromatography. Lane M represented the protein molecular marker (kDa), while Lanes 1 to 7 were sequentially eluted with 60 mM imidazole and NaCl concentrations of 50, 200, 500, 800, 1200, and 4000 mM.


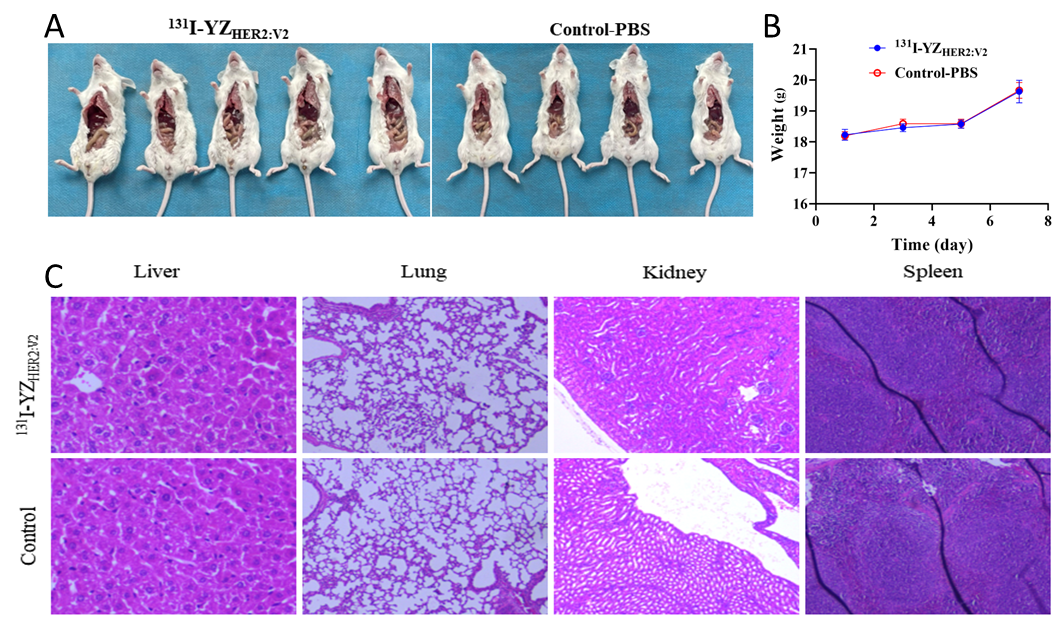


**Figure S3.** ^131^I-YZ_HER2:V2_ abnormal toxicity assay. A. After dissection, there was no significant difference in the morphology and color of organs between the experimental group and the control group. There was no significant difference in body weight between the experimental group and the control group (the first day: 18.23±0.17 in the experimental group, 18.19±0.13 in the control group, P=0.75; On the third day, the experimental group was 18.46±0.12, the control group was 18.58±0.14, P=0.20; On the 5th day, the experimental group was 18.57±0.13, the control group was 18.58±0.14, P =0.921; 7 d: 19.62±0.36 in the experimental group and 19.66±0.25 in the control group,P=0.87). C, Hematoxylin-eosin staining showed that the structure of the cells was intact under the microscope, and there was no significant difference between the two groups.


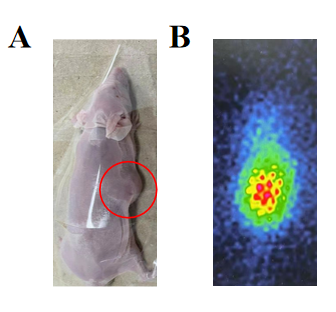


**Figure S4**. SPECT static imaging of an ID8 ovarian cancer nude mouse model. A. ID8 ovarian cancer nude mouse model; B. SPECT static imaging showed no radioactive uptake in the tumor
